# Design and pilot evaluation of an evidence-based worksheet and clinician guide to facilitate nutrition counselling for patients with severe mental illness

**DOI:** 10.1186/s12888-021-03575-7

**Published:** 2021-11-10

**Authors:** Laura LaChance, Monique Aucoin, Kieran Cooley

**Affiliations:** 1 McGill University, Department of Psychiatry, Ludmer Research & Training Building, 1033 Pine Avenue West, Montreal, QC H3A 1A1 Canada; 2 St. Mary’s Hospital Centre, 3830 Lacombe Avenue, Montreal, QC H3T 1M5 Canada; 3 Canadian College of Naturopathic Medicine, 1255 Sheppard Ave E, North York, ON M2K 1E2 Canada; 4 University of Technology Sydney, Ultimo, Australia; 5 Pacific College of Health Sciences, San Diego, USA; 6 National Centre for Naturopathic Medicine, Southern Cross University, Lismore, Australia

**Keywords:** Diet, Nutrition, Worksheet, Psychosis, Schizophrenia, Counseling, Lifestyle, Knowledge translation, Clinical tool, Patient education

## Abstract

**Background:**

Schizophrenia spectrum disorders (SSD) are severe, persistent mental illnesses resulting in considerable disability and premature mortality. Emerging evidence suggests that diet may be a modifiable risk factor in mental illness; however, use of nutritional counselling as a component of psychiatric clinical practice is limited. The objective of this project is the design and evaluate a worksheet and clinician guide for use in facilitating nutritional counseling in the context of existing mental health care.

**Methods:**

The worksheet and clinician guide were developed based on the results of a recent scoping review on the relationship between diet and mental health symptoms among individuals with SSD. A feedback process involved a focus group with psychiatrists and interviews with individuals with lived experience with psychosis. Participants were asked a series of structured and open-ended questions. Interviews were transcribed and data units were allocated to categories from an existing framework. The comments were used to guide modifications to the worksheet and clinician guide. A brief interview with all participants was completed to gather feedback on the final version.

**Results:**

Five psychiatrist participants and six participants with lived experience completed interviews. Participants provided positive comments related to the worksheet design, complexity and inclusion of interactive components. A novel theme emerged relating to the lack of nutritional counselling in psychiatric training and clinical practice. Many constructive comments were provided which resulted in meaningful revisions and improvements to the worksheet and clinician guide design and content. All participants were satisfied with the final versions.

**Conclusions:**

A worksheet and clinician guide designed to facilitate nutritional counselling with individuals with SSD was found to be acceptable to all participants following a process of feedback and revision. Further research and dissemination efforts aimed at increasing the use of nutritional counselling in psychiatric practice are warranted.

**Supplementary Information:**

The online version contains supplementary material available at 10.1186/s12888-021-03575-7.

## Background

Schizophrenia spectrum disorders (SSD) are a group of severe, persistent mental illnesses including schizophrenia, schizoaffective disorder and other psychotic disorders. Mental health symptoms include positive symptoms such as delusions or hallucinations, negative symptoms such as low motivation and social withdrawal and cognitive symptoms. In addition to considerable disability associated with mental health, this population suffers from elevated rates of medical comorbidities and premature mortality as compared to the general population [1]. Antipsychotic medications (in addition to psychosocial interventions) are a mainstay of treatment though these medications contribute to metabolic dysregulation already present at the onset of SSD [2–4]. Many environmental factors also contribute to the differences in rates of chronic disease including socioeconomic status, low levels of physical activity [5], poor diet [6], and elevated rates of smoking [7]. Including interventions to target health behaviours such as diet in the care of individuals with SSD to address excess morbidity and mortality due to physical health conditions is recommended for individuals with SSD though uptake of such interventions is low [8].

An emerging area of literature suggests that nutrition is an important modifiable risk factor in the development and progression of mental illness [9]. Observational data show strong correlations between poorer diet and poorer mental health [10, 11]. More recently, experimental studies have demonstrated the potential for diet interventions to have a significant adjunctive treatment effect for mental illness [12]. An extensive scoping review identified all of the published literature related to the relationship between diet and psychiatric symptoms in SSD [13]. This review synthesized the results of 822 articles which reported on a range of diet patterns and food constituents. Observational studies reported an association between the presence of SSD and lower quality diet patterns, higher refined carbohydrate and total fat intake and lower levels or intake of omega-3 and omega-6 fatty acids, dietary fibre, vegetables and fruit and several vitamins and minerals including folate, zinc, vitamin B12 and B6, vitamin C, and selenium. Microbiome composition, food allergy and sensitivity and intake of phytonutrients such as L-theanine, sulphorphane and resveratrol are associated with onset and progression of psychotic disorders. A number of experimental studies have been conducted in which diet or nutrient interventions were provided to patients with SSD. Benefits to mental health symptoms have been documented as a result of interventions such as healthy diet patterns, vitamin and mineral supplementation (vitamin B12 and B6, folate, zinc) and amino acid supplementation (serine, lysine, glycine and tryptophan) [13].

The use of limited amounts of nutrition counselling in medicine as a whole has been identified. In a study directly observing practice behaviour of American family doctors, nutritional counselling took place in about one quarter of patient encounters and the average amount of time spent was 55 s [14]. Nutrition guidelines [15] and tools [16] exist to assist patients in the management of medication-induced weight gain and physical illness. However, there is very limited evidence of use of nutritional strategies in the management of mental health symptoms. While the dietary strategies indicated for the management of physical comorbidities [17] and the management of psychiatric symptoms have significant overlap, nutritional strategies that are not indicated in the management of cardiometabolic illness were identified as being potentially useful for the management of psychiatric symptoms in the recent review [13]. The focus of the present tool is primarily the addition of nutritious foods rather than the restriction of calories or individual foods. In order achieve this goal, the tool should not contradict generally accepted recommendations for weight management, it should also suitable for patients who have a low or normal body mass index. Additionally, the tool should provide psychoeducation about the importance of quality nutrition as a determinant of mental health. There have been calls for increased use of nutrition in the treatment of psychiatric disorders [18, 19]; however, it is recognized that medical doctors [20], including psychiatrists, and other mental health professionals receive limited training in nutrition [21] and that additional training and resources are needed.

Printed educational materials are an established knowledge translation strategy to narrow the research to practice gap [22]. This low-cost strategy is useful for increasing clinician knowledge, motivation and behaviour according to a Cochrane review assessing the effectiveness of these types of clinical tools. In the current project, the clinician behaviour of interest is engaging psychiatric patients in a conversation about the role of food in mental health in addition their usual care provided.

The objective of this knowledge translation project was to synthesize the results of a previously published scoping review on the relationship between diet and mental health symptoms in SSD into an evidence-based worksheet and accompanying clinician guide. In order to ensure the worksheet and clinician guide were acceptable and appropriate for our target audience of mental health professionals and individuals with severe mental illness, we aimed to complete an evaluation and revision process involving clinicians and individuals with lived experience.

## Methods

### Worksheet and clinician guide design

The first draft of the worksheet was developed by the research team and included diet recommendations that were most supported by the evidence identified in the scoping review [13] and consistent with Canada’s Food Guide [23]. The worksheet development was guided by the Social Cognitive Theory which highlights the important roles of goal setting and behaviour contracting, reinforcement, self-control, social norms, attitudes, and self-efficacy [24]. The worksheet provides basic education about important nutrition principles and very simple recommendations to increase knowledge about healthy eating and clear actionable tips for individuals to incorporate. It aims to maintain a positive tone. Motivational interviewing questions were included to increase motivation, self-reflection, and goal setting. Delivery of the intervention by the patient’s mental health care provider is by design, in an attempt to address the widely held misbelief that nutrition intervention is of limited importance to mental health care and begin to change norms. Given the high rates of poverty and food insecurity in individuals with SSD and other severe mental illnesses [25], it is commonly suggested that eating a nutritious diet is prohibitively expensive for this population. This barrier to healthy eating is directly addressed in the worksheet in a section related to eating well on a budget. Here, the worksheet offers practical tips to increase perceived control and self-efficacy. In accordance with recognized best practices, the worksheet attempted to keep a friendly and encouraging tone, maintain a clear and easy to read format, and use bullets, tables, short paragraphs, non-technical language, and a 5th grade reading level [26, 27]. Efforts were made to include foods from a range of cultural backgrounds. Lastly, previous research on implementing diet changes in a psychosis population was considered [28, 29]. In addition to financial costs, other barriers to uptake of nutritional interventions in this population include cognitive and knowledge deficits, social isolation, decreased motivation and the effects of antipsychotic medications on appetite and metabolic regulation. Previous studies have identified practical strategies for overcoming these obstacles and have demonstrated success in doing so [30] and these were considered in the worksheet design.

The initial draft of the clinician guide included background information as well as the worksheet goals and target population. It included a suggested agenda for reviewing the worksheet in a clinical encounter and scientific rationale for the nutrition recommendations provided in the worksheet.

### Pilot testing

In order to ensure that this worksheet and clinician guide met the needs of our target population, we undertook a process to evaluate the worksheet using established, pre-identified criteria: interest, informativeness, ease of understanding, visual appeal, trustworthiness, usefulness and degree of encouragement [27]. The pilot testing took place in the form of a 75-min virtual focus group (licensed version of Zoom) involving psychiatrists (*n* = 5) and a series of 15 to 30-min individual phone interviews with individuals with lived experience of psychosis (*n* = 6). The sessions took place during September and October of 2020. Participants were recruited through announcements at staff meetings, posters displayed in clinical areas and flyers distributed to clinicians in the mental health department at St. Mary’s Hospital Centre in Montreal, Canada. Some patient participants self-referred to the study. In other cases, the patient’s psychiatrist brought the study to the patient’s attention and requested consent for the patient to be contacted by the study team. Determination of sample size was guided by a recent publication relating to qualitative research power [31]. Factors considered in its determination included a narrowly focused question of assessing the tool, the abundance of existing theory on dietary guidelines and behaviour change and the needs of patients with psychotic disorders. We anticipated that clinicians would be able to clearly communicate their opinions on the topic and that although people with lived experience may be able to communicate less clearly, this would be off-set by the use of focused questions. Considering these factors, we aimed to recruit six to eight individuals with lived experience and four to six clinicians.

The psychiatrists were required to meet the following inclusion criteria: 1) Currently in active medical practice in Quebec 2) Treat patients suffering from psychotic disorders on a regular basis (at least monthly) 3) Able to provide informed consent 4) No formal nutrition training. The participants with lived experience were required to meet the following criteria: 1) Self-report of current or previous psychotic disorder OR friend/family member of an individual with a past or current psychotic illness 2) Clinically stable, measured by no change in psychiatric medication in the previous four weeks 3) Able to read in English and provide informed consent. While no limitations were placed on the duration of psychotic illness, we attempted to recruit individuals with varying duration of illness in order to reflect the population of patients who will be candidates for incorporating the worksheet as part of their clinical care.

All participants provided informed consent prior to data collection. The sessions included a structured component in which participants rated the worksheet on whether they perceived it to be interesting, informative, trustworthy, easy to understand, useful, attractive and encouraging using a three-point scale (‘extremely’ ‘somewhat’ and ‘not at all’) [27]. The interviews also included a series of open-ended questions that were used to generate discussion and gather qualitative feedback [16]. These questions included:

1. What are some of your thoughts about using this worksheet to learn about and discuss healthier diet choices?
2. What part of the worksheet do you find most helpful?
3. What challenges or obstacles do you see that might prevent you from using the worksheet?
4. Do you have recommendations that would help make the worksheet more useful?
5. Are there other things you would like to say before we close the session?

Key areas of discussion were incorporated into subsequent interviews in manner consistent with iterative qualitative inquiry. All participants completed a nutritional literacy assessment, the Newest Vital Sign (NVS) [32]. The maximum score of the NVS is 5 and a score of 4 or 5 suggests adequate literacy. A score of 2 or 3 suggests limited literacy. The individuals with lived experience completed a demographics questionnaire, anonymously.

### Data analysis and revision

All interviews were audio recorded with participant permission and transcribed verbatim. Data analysis consisted of four steps: 1) identification of units of analysis in the interview transcripts, 2) allocation of the data units to categories, 3) counting and interpretation, 4) revision to the worksheet. The a priori categories used were: 1) Content/Information 2) Complexity 3) Structure/Orientation 4) Layout/Design and 5) Patient Centeredness. Comments that did not fit within one of the predefined categories were documented separately. This framework was adapted from previously completed research using qualitative feedback to improve a patient education worksheet [33]. Review of the comments that did not fit in the predefined categories resulted in the creation of an additional category that was added to the existing framework. The sixth category was called “Nutrition as a blind spot”. Comments relating to the worksheet and the clinician guide were analyzed separately. Identification of data units was completed by two members of the research team (LL and MA) independently. Subsequently, two researchers (LL and MA) separately categorized each statement to facilitate reliability and rigor and consensus was used to resolve any disagreements. Interpretation and revisions to the worksheet draft took place through a discussion.

### Pilot testing, second round

The revised version of the worksheet was mailed to all participants and a brief 10-min phone interview was completed to allow participants to provide feedback on the revised version. Two attempts were made to contact each participant.

### Ethics

The Research Ethics Boards of St. Mary’s Hospital Centre (CIUSSS de l’ouest de l’ile de Montreal) and the Canadian College of Naturopathic Medicine provided oversight to this project.

## Results

**Fig. 1:**
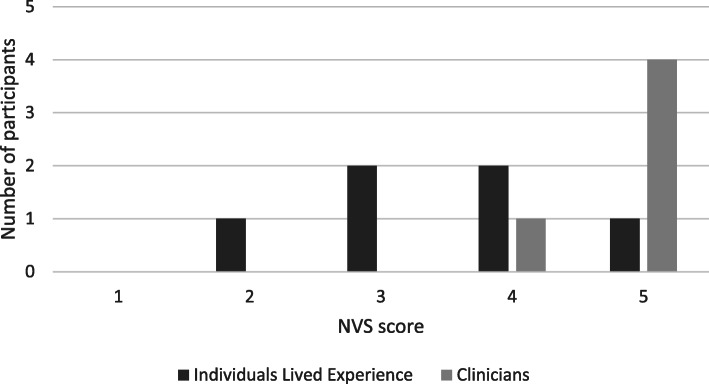
Score on the Newest Vital Sign (NVS) nutrition literacy assessment tool. The maximum score is 5 and a score of 4 or 5 suggests adequate literacy. A score of 2 or 3 suggests limited literacy

The first draft of the worksheet and clinician guide were developed by the team of investigators (Supplementary file 1) and sent to all participants. Individual interviews were completed with six participants with lived experience. All participants with lived experience were under the care of a psychiatrist at St. Mary’s Hospital Centre. Specific diagnoses were not confirmed by the study team. No family members of individuals with lived experience with psychosis were recruited. The sample included male and female participants who were diverse in their age and illness duration (Table 1). The focus group was attended by five clinicians, all practicing psychiatrists employed by St. Mary’s Hospital Centre in Montreal, Quebec, Canada. Participants’ scores on the Newest Vital Sign nutrition literacy assessment tool are displayed in Fig. 1. All of the clinicians scored in the “adequate literacy” range while half of the participants with lived experience scored in the “limited literacy” range.

**Table 1:** Demographic and clinical information for Individuals with Lived Experience (*n* = 6)

Characteristic	Number of participants
**Demographic**	
Gender	
Male	3
Female	3
Age	
< 20	1
21–30	1
31–40	1
41–50	1
51–60	1
> 60	1
Financial Support	
Employed	1
Government support	3
Family support	2
**Clinical**	
Illness Duration	
< 5 years	2
> 5 years	4

Participant responses to the structured questions about the worksheet are displayed in Table 2. The responses were primarily positive. Participants with lived experience consistently rated the worksheet as useful, informative, and easy to understand but only somewhat attractive. The clinician participants rated the worksheet as highly interesting and informative but only somewhat useful, attractive, trustworthy, and easy to understand. Table 2 also displays the responses provided by the clinicians about the clinician guide. The clinicians rated the guide as extremely interesting, informative, and easy to understand but somewhat attractive.

**Table 2:** Responses to structured questions about the worksheet and clinician guide from individuals with lived experience and clinicians

	Worksheet, Individuals with lived experience			Worksheet, Clinicians			Clinician Guide		
Structured Question	Extremely	Somewhat	Not at all	Extremely	Somewhat	Not at all	Extremely	Somewhat	Not at all
Interesting	4	2	0	5	0	0	5	0	0
Informative	6	0	0	4	1	0	4	1	0
Trustworthy	3	3	0	2	3	0	3	2	0
Easy to Understand	6	0	0	1	4	0	2	1	0
Useful	6	0	0	1	4	0	3	2	0
Attractive	2	4	0	2	3	0	1	5	0
Encouraging	4	2	0	3	2	0	3	2	0

Data units were allocated to one of six categories. Based on the constructive data points, actions to revise the worksheet and clinician guide were established. See Table 3 for a summary of themes and relevant revisions that were undertaken to modify the first draft of the worksheet and clinician guide.

**Table 3:** Number of data units allocated to each category, summary of themes and revisions completed

	Number of data points (n positive, n constructive)	Positive themes (frequency)	Constructive themes (frequency)	Revisions based on data
Worksheet				
Content/ Information	28 (14 positive, 14 constructive)	-appreciated the worksheet (3), the first page of recommendations (6), table on cost (1), healthy dessert ideas (1), complex carb recommendations (1), sample plate (1) -appreciated inclusion of motivational interviewing components (1)	-more sample meals (3) -more transparency on the cost of food (1) -link to other resources about minimizing cost (1) -increase relative content of motivational enhancement material (1) -remove scoping review reference (1) -more guidance on portion sizes (5) -include plant-based options (1) -consider adding a shopping list (1)	-second day of meal examples added -scoping review reference removed -additional resources related to cost and portion size added to clinician guide -suggestions such as the addition of portion size information to the worksheet were not acted on as the authors felt they would increase the complexity and length of the worksheet substantially
Complexity	9 (4 positive, 5 constructive)	-complexity was appropriate (4)	-recommendations must be specific and easy to implement (2) -6th grade reading level (1) -fewer words to read (1) -more pictures to assist individuals with language barriers or cognitive deficits (1)	- removed vague or theoretical recommendations, improved congruence with 5th grade reading levels and minimize text
Structure	6 (2 positive, 4 /constructive)	-interactive nature of the worksheet was appreciated (2)	-move motivational enhancement material earlier in the worksheet (3) -consider flier format (1)	-Question about personal importance of healthy eating moved to the top of first page
Design	16 (3 positive, 13 constructive)	- overall design (1) and plate image (2) was appreciated	-improve plate recognizability (2) -consider adding colour (3) -more distinction between educational material and action items (1) -more pictures of example foods (2) -include pictures of foods to avoid (5)	-cutlery added to plate image to increase recognizability -boxes placed around the activities for participants to complete in order to increase recognition -colour was not added as printing a colour version in black and white would result in lower quality images -foods to avoid were not added as the worksheet attempted to maintain s focus on positive messaging
Patient Centeredness	10 (1 positive, 9 constructive)	-recommendations are accessible and feasible (1)	-importance of cultural sensitivity (5) -importance of financial considerations (2) -accessibility in different languages (2)	-included food recommendations from a larger number of cultures as well as low-cost options -future plan to translate into French and other languages
Clinician Guide				
Content/ Information	14 (2 positive, 12 constructive)	-appreciated having the guidance document	-increase clarity about the level of evidence supporting the statements in the clinician guide (6) -remove material that is redundant with the worksheet (1) -clarify mechanism of action information in chart (1) -remove reference to patient contract to reduce ambiguity (1) -increase the number of sample MI questions (1) -consider adding more references to support the recommendations (3)	-The “rationale” column was split into two columns: “supporting evidence” and “mechanism of action” -Levels of evidence were added -The level of detail for the mechanisms of action were reviewed to ensure that it was appropriate -recommendations that were also found in the worksheet were removed from the clinician guide -reference to contracting was removed -additional references were not added in order to avoid lengthening the guide; clinicians are directed to the scoping review to access more information about the research used to create the recommendations
Complexity	0			
Structure	1 (constructive)		-consider lengthening guide beyond 1 page (double sided) to allow for additional detail to be included	-guide lengthened to 3 pages to allow for additional detail to be included and to increase visual appeal
Design	0			
Patient Centeredness	21 (2 positive, 19 constructive)	-participants appreciated cultural sensitivity (1) and efforts to include an interdisciplinary team in the patient’s care	-lack of guidance on next steps if a patient declines to discuss their diet (1) -lack of clear statement related to the gradual nature of diet behaviour change and expectations for the initial session (5) -suggestion to include assessment of patient diet and tailored recommendations (3) -many patients have limited food budgets (5) -making dietary changes is difficult (4) -untreated psychosis may interfere with patients’ ability to engage in dietary counselling (1)	-recommendation for how to approach patient refusal was added -statement added about the gradual nature of diet change -while comprehensive diet assessment was deemed beyond the scope of the worksheet, optional instructions were added to the guide for interested clinicians -revisions were made to the clinician guide to emphasize the healthy foods that could be purchased for $10 and recommendations were made for approaching food insecurity if identified -more specific guidance about motivational enhancement was added -included instruction to use the worksheet during periods of relative clinical stability

A noteworthy theme that emerged from the discussion with both groups of participants was the lack of nutrition training in medical education and psychiatric residency and the general absence of nutritional counselling in this field of medicine. This category was named “Nutrition as a blind spot” and included 15 data units. One participant with lived experience stated “I think that it [nutrition] has definitely a place in psychosis and in schizophrenia, definitely an unspoken area. It doesn’t get talked about at all. Especially in institutionalized settings. You are overloaded with sugars, like not healthy grain, not complex grain, you get white bread, sandwiches, shitty juice”, while another reported “My only doctor is my psychiatrist, so basically I’m on my own to decide what to eat. I could envision this worksheet to facilitate a conversation with my psychiatrist.” One psychiatrist reported “I admit that I only sometimes asked them …. What they …. not even what they eat, but IF they eat”, while another stated “If you’re getting clinicians to think about this, it is sort of raising our vigilance in the patient encounter, which is not just looking at our usual, traditional medical focus, but really looking at their lifestyle. We do talk about medications and weight gain, and metabolic syndrome. And we’re good at testing it. I think we’re getting better at exercise [ …] but nutrition, I’d say, is really a blind spot.” While the data within this category were not used to modify the worksheet, this finding reinforces the need for the present research efforts to facilitate initial dietary counselling as part of mental health care for individuals with severe mental illness.

The worksheet and clinician guide were modified based on participant feedback to create the revised versions (Supplementary file 2 and 3). The French versions of the tool and clinician guide are available as Related files 1 and 2. Brief phone calls with the participants with lived experience (4/6), and clinicians (5/5) indicated that they were satisfied with the changes made and felt that the final product adequately met their needs. No additional concerns or points of criticism were raised.

## Discussion

The present publication describes the development and evaluation of a nutrition education worksheet and clinician guide which disseminate the findings of a scoping review on the relationship between diet and mental health symptoms in psychotic disorders. Pilot testing through interviews with clinician participants (psychiatrists) and participants with lived experience resulted in favourable ratings in many domains and many positive comments about the initial draft. Participants also raised several concerns and provided constructive suggestions for improvement which were incorporated into the final version. The final version of the worksheet and clinician guide were found to be acceptable to all study participants.

With respect to the target population for this worksheet, it is noted that the scoping review that informed the development of the worksheet and clinician guide was based on studies of participants with psychotic disorders. As such, the dietary recommendations provided in the worksheet are specific to this population. Given the significant overlap in clinical presentation, pathophysiology and management of severe mental illnesses, and the worksheet’s consistency with broadly accepted nutrition principles, delivery of this intervention to a wider population of individuals with severe mental illness may be considered.

This project has a number of strengths. The worksheet and clinician guide are a novel approach to integrating nutrition counseling within mental health treatment as usual. The methods included an assessment of nutritional literacy and the qualitative analysis was guided by an existing framework developed for evaluation of a patient education tool. The study included participants with lived experience, who were diverse with respect to age and illness duration, as well as clinician participants from our target population for tool delivery. Participants with lived experienced reported that the level of complexity and understandability of the worksheet was at an appropriate level despite half of participants with lived experience demonstrating limited nutrition literacy.

Limitations of the project include a convenience sample of participants from a single hospital setting. St. Mary’s Hospital Centre is a general hospital within the McGill University network. The psychiatry department offers second line mental health services to a general adult psychiatry population that is ethnically diverse. Clinicians in other practice settings may have reported different feedback about the worksheet and clinician guide; however, the population of psychiatrist participants is thought to be representative of general adult psychiatrists practicing in a busy urban hospital setting. We elected to focus our clinician sample on psychiatrists because we considered this group the primary target for this knowledge translation intervention. While providers such as dieticians and social workers are generally more likely to assess lifestyle factors, assessment, and modification of diet by psychiatrists is very limited based on the authors’ personal experience working and teaching in two Canadian major academic centres, and through experience presenting about nutrition and mental health at national and international psychiatry conferences. Survey data assessing the use of nutrition counselling in North American psychiatry practice is not available. It is noted that the unsatisfactory use of nutrition counselling in psychiatry was a theme presented by numerous participants in the present study, both clinicians and individuals with lived experience, adding evidence to the current lack of use and need for targeted knowledge translation efforts.

Additionally, the sample size may be considered a limitation of the present project. We did not recruit any family members of patients with lived experience. This may have been influenced by restrictions on family members or caregivers entering the hospital during the COVID-19 pandemic in that there was a lack of opportunity for family members to encounter the study flyers in the waiting room or discuss the study with their family member’s psychiatrist. While the participants with lived experience who participated in the interviews were diverse in many respects (age, gender, income, duration of illness), we did not query participants about their ethno-racial background. It is unclear if the participants were fully representative of the wider community of individuals with SSD. Efforts were made to include sample foods from a range of cultures; however it should also be acknowledged that access to mental health services can vary amongst ethnic populations in Canada [34]. Because the authors aimed to limit the length of the tool to one double-sided page, there was not sufficient space to provide meal plans tailored to individual cultures. Subsequent work in this area could include the design and pilot testing of nutritional resources (e.g. handouts, web-applications) which might support a wider array of cultures or dietary diversity. Additionally, rigorous evaluation of the tool in a large sample of participants who represent a range of ethno-racial backgrounds is indicated.

Another limitation of the project is the passive nature of the worksheet format as a knowledge translation strategy. While printed educational materials have documented effects on clinician behaviour change, these changes may be limited in magnitude [22]. Additional strategies will likely be necessary to increase the likelihood of uptake among clinicians. These could include presentations at educational meetings involving active audience participation and skill building [35], development of continuing education courses [35], dissemination within communities of practice [36] and social media [37], and promotion by local opinion leaders [38]. It is noted that passive dissemination strategies, such as printed educational materials, are more likely to change clinician behaviour when there is existing motivation [39]. In response to a recent survey of mental health professionals from 52 countries, 92% of respondents indicated that they were interested in expanding their knowledge of nutrition as a therapeutic approach to mental health care [21]. This high level of motivation suggests that the dissemination strategy is appropriate.

Furthermore, clinical interventions providing educational materials alone may have a limited impact on patient behaviour change. This is acknowledged as a limitation of the present intervention. The development of the worksheet was guided by the Social Cognitive Theory with an emphasis on increasing patient knowledge of nutrition and the benefits of diet change, increasing motivation and minimizing barriers to change. Substantial efforts have been made to include motivational enhancement and interactive components in the worksheet design such as goal setting. Given the nature of a worksheet, it was not possible to include activities for skill building or observational learning; however, the clinician guide provides recommendations for connecting the patient with members of an interdisciplinary team to support further dietary intervention and acquisition of the relevant skills and resources. For example, a nutrition professional such as a dietician, nutritionist, or a naturopath, could provide patients with more individualized dietary recommendations and address gaps in skills. Social workers could facilitate increased access to healthy foods via food banks, government programs or other community resources. Occupational therapists could support patients in making dietary changes by working in a behavioural activation, cognitive adaptation, or goal-setting framework. Overall, mental health professionals, such as our study population - psychiatrists, are well-trained in supporting behaviour change but nutritional training is lacking [21]. Future work with other mental health care providers to introduce, modify, or implement the tool may be valuable. The present worksheet and clinician guide are meant to provide some initial education and guidance to increase mental health professionals’ confidence and competence in the delivery of dietary interventions as part of a treatment plan for individuals with psychotic disorders.

The worksheet and clinician guide are not meant to replace consultation with a nutrition professional but rather to help facilitate an initial dietary intervention within the patient’s established clinical relationship with a mental health professional. Doctors are uniquely positioned to influence public perception about the importance of lifestyle factors. For instance, even very brief interventions involving a simple series of questions delivered by MDs have demonstrated efficacy on smoking cessation rates [40]. This finding has led to widespread education about smoking cessation in medical school through concepts such as the “5 A’s” strategy for smoking cessation [41]. The simple act of engaging with patients about the important role that food plays in mental health, within the context of usual care, reinforces the importance of dietary change in this population. One patient interaction at a time, clinicians can start to shift norms in this area in a way that is in line with the increasing awareness of the importance of nutrition for health and need for nutritional education and counselling, within psychiatry and the broader medical field [9, 42, 43]. Future studies could evaluate the impact of this clinical worksheet on clinician and patient attitudes, knowledge, and behaviour related to nutrition counselling in the context of mental health care provision or tailor the design to the unique needs of different ethno-racial populations.

## Conclusions

This publication presents an evidence-based worksheet and clinician guide to support mental health professionals to engage patients with severe mental illness in basic dietary counseling. A feedback process involving practicing psychiatrists and individuals with lived experience of psychosis was undertaken; many constructive comments resulted in meaningful revisions and improvements to the design and content. All participants provided positive comments, recognized a need for research in this area and were satisfied with the final version. Further research on this worksheet is warranted, including evaluation with a larger sample size.

## Supplementary Information

**Additional file 1. Supplementary file 1:** Eating well for mental health, draft worksheet.

**Additional file 2. Supplementary file 2:** Eating well for mental health, revised worksheet.

**Additional file 3. Supplementary file 3:** Revised clinician guide for Eating well for mental health worksheet.

## Data Availability

The datasets used and/or analyzed during the current study are available from the corresponding author on reasonable request.

## Abbreviations

SSD: Schizophrenia spectrum disorders
