# Supplementary material for: Design and pilot evaluation of an evidence-based worksheet and clinician guide to facilitate nutrition counselling for patients with severe mental illness
Source: BMC Psychiatry. 2021 Nov 10;21:556. doi: 10.1186/s12888-021-03575-7 (PMC8579166; doi:10.1186/s12888-021-03575-7)
Supplement: Supplementary file 1 — Additional file 1. Supplementary file 1: Eating well for mental health, draft worksheet. [file 12888_2021_3575_MOESM1_ESM.pdf]

Eating well for mental health

A tool to support people affected by severe mental illness

The food we eat affects our body and mind. You can make healthier choices that will improve your health. Circle ideas that appeal to you.

DRAFT

Choose complex carbohydrates

- Whole grain bread or pasta instead of white
- Instead of white rice, try brown rice, wild rice, quinoa, oats
- Vegetables like potatoes, sweet potatoes, corn, squash

Choose healthy fats and sources of omega-3

- Eat fish and seafood (oysters, mussels, shrimp) at least 3 times per week
- Add nuts and seeds to meals or enjoy as a snack
- Decrease deep fried foods (French fries, fried chicken)

Reduce highly processed foods and sugar

- Drink water as your main beverage instead of pop, juice and iced-tea
- Have pleasure foods like cookies, cakes, muffins, ice cream, candy as a special treat, not every day

Include vegetables & fruits with meals and snacks

- Increase amount and variety - try to eat the rainbow
- Try salad, veggies and dip, frozen vegetables added to meals
- Try fruit that is fresh, frozen or canned as a snack or dessert

Eat protein-rich foods throughout the day

- Try fish, seafood, chicken, turkey, beans, lentils, nuts, seeds, soy, eggs, and meat

Aucoin M, LaChance L, Cooley K, Kidd S. Diet and Psychosis: A Scoping Review Neuropsychobiology. 2018 Oct 25:1-23.

McGill

CCNM
Canadian College of Naturopathic Medicine

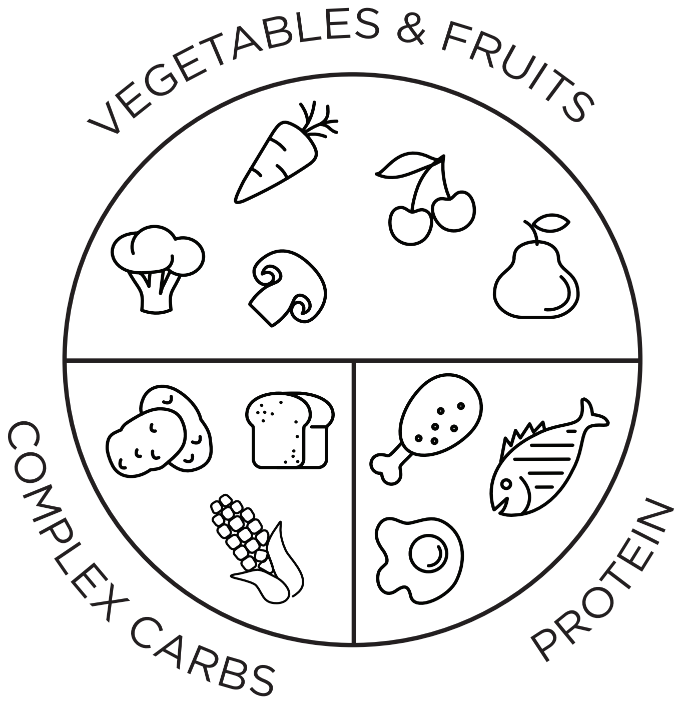

Example meals:

Breakfast

- Oatmeal with nuts/seeds and berries or banana
- Eggs with fruit and whole grain toast or potatoes

Lunch or Dinner

- Salad with protein-rich food (ex: chickpeas, hard-boiled egg, canned salmon) and healthy fat (ex: nuts, avocado, olive oil)
- Whole grain pasta with chicken, vegetables, and your favourite sauce

Snack or Dessert

- Vegetables with hummus
- Apple with peanut butter, Greek yogurt with fruit

I'm going to try / Personal example / I might like:

____

Healthy foods that are not expensive:

Tip: Choose foods in season, shop at budget grocery chains, avoid convenience stores, look for sales and stock up on non-perishable foods

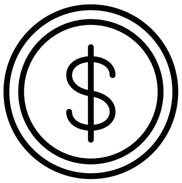

Frozen vegetables and fruit	$3-4 per bag (4 or more servings)
Canned fish and seafood (tuna, oysters, salmon)	$1-3 per can (1 serving)
Canned beans and lentils	$1 per can (2 servings)
Eggs	$2-3 per dozen (6 servings)
Grains in large packages (brown rice, oats, whole grain pasta)	$3-5 per bag (5 or more servings)

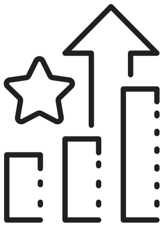

My goals

Habit I would like to continue: ____________________________

Changes I would like to make: ____________________________

Who can help me achieve these goals? ________________________

Why healthy eating is important to me? ________________________

Preparing food for yourself and making healthy dietary choices is self-care. Small changes add up. Share this activity with others as a way to support each other and improve well-being.

DRAFT
