# Supplementary material for: Design and pilot evaluation of an evidence-based worksheet and clinician guide to facilitate nutrition counselling for patients with severe mental illness
Source: BMC Psychiatry. 2021 Nov 10;21:556. doi: 10.1186/s12888-021-03575-7 (PMC8579166; doi:10.1186/s12888-021-03575-7)
Supplement: Supplementary file 2 — Additional file 2. Supplementary file 2: Eating well for mental health, revised worksheet. [file 12888_2021_3575_MOESM2_ESM.pdf]

Eating well for mental health

A tool to support people impacted by severe mental illness

The food we eat affects our body and mind. You can make healthier choices that will improve your health.

Why is healthy eating important to me? ______________________________

Read the recommendations below. Circle foods that appeal to you.

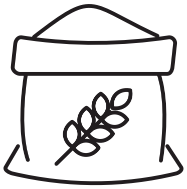

Choose complex carbs

- Whole grain bread, pita, tortilla, or pasta instead of white
- Instead of white rice, try brown rice, wild rice, quinoa, oats, millet
- Vegetables like potatoes, sweet potatoes, yams, corn, squash, cassava

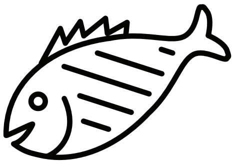

Choose healthy fats like olive oil and sources of omega-3

- Eat fish and seafood (oysters, mussels, shrimp) at least 3 times per week
- Add nuts, seeds or avocado to meals, or enjoy as a snack
- Decrease deep fried foods (French fries, fried chicken)

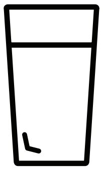

Reduce highly processed foods and sugar

- Drink water instead of pop, juice and iced-tea
- Have cookies, cakes, muffins, ice cream, candy as a special treat, not every day

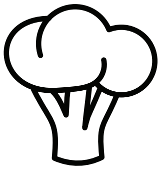

Add vegetables & fruits to meals and snacks

- Try to include different colours
- Add salad or veggies and dip to meals. Explore frozen and canned vegetables
- Try fruit that is fresh or frozen as a snack or dessert

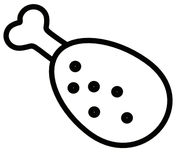

Eat protein-rich foods throughout the day

- Try eggs, fish, seafood, chicken, turkey, beans, lentils, nuts, seeds, tofu, cheese, meats and wild game

McGill

CCNM
Canadian College of Naturopathic Medicine

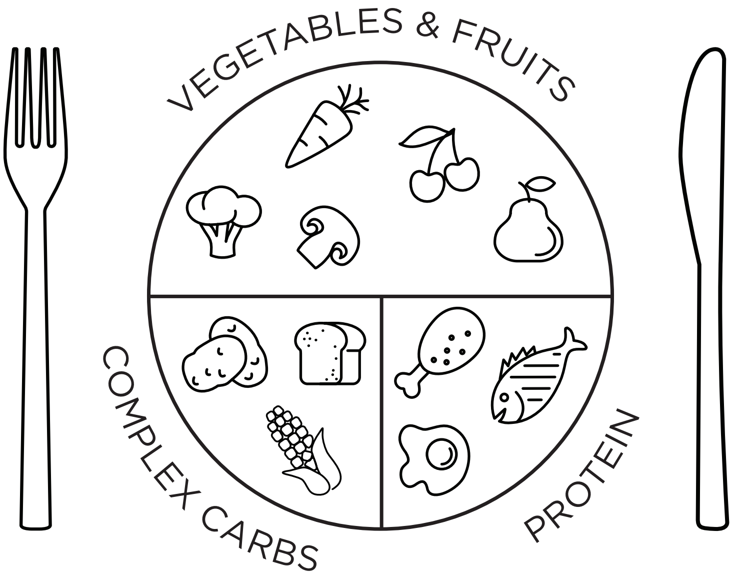

Two Day Sample Meal Plan:

	Day 1	Day 2
**Breakfast**	Oatmeal/ porridge with nuts or seeds and berries	Eggs with whole grain toast or potatoes and fruit
**Lunch**	Salad with protein-rich food and healthy fat	Turkey sandwich with side of sliced cucumbers and carrots
**Dinner**	Whole grain pasta with tomato sauce and added ground chicken and vegetables	Brown rice stir-fry with shrimp or tofu and frozen vegetables
**Snack or Dessert**	Vegetable sticks with hummus or guacamole	Plain yogurt with fruit

I'm going to try / I might like:

____

Healthy eating doesn't need to cost more:

Lower Cost Healthy Foods	Price (in Canadian dollars)
Frozen vegetables and fruit	$3-4 per bag (4 or more servings)
Canned fish and seafood (tuna, oysters, salmon, mussels, sardines)	$1-3 per can (1 serving)
Canned beans and lentils	$1 per can (2 servings)
Eggs	$2-3 per dozen (6 servings)
Whole grains in large packages	$3-5 per bag (5 or more servings)
Tofu	$3 per pacakge (4 servings)

Tip: Shop at budget grocery stores, look for sales and stock up on non-perishable foods

My goals

Habit I would like to continue: ______________________________

Changes I would like to make: ______________________________

Who can help me achieve these goals? ______________________________

Preparing food for yourself and making healthy food choices is self-care. Small changes add up. Eat and prepare meals with others.
