# Supplementary material for: Design and pilot evaluation of an evidence-based worksheet and clinician guide to facilitate nutrition counselling for patients with severe mental illness
Source: BMC Psychiatry. 2021 Nov 10;21:556. doi: 10.1186/s12888-021-03575-7 (PMC8579166; doi:10.1186/s12888-021-03575-7)
Supplement: Supplementary file 3 — Additional file 3. Supplementary file 3: Revised clinician guide for Eating well for mental health worksheet. [file 12888_2021_3575_MOESM3_ESM.pdf]

Eating well for mental health
Clinician Guide

Overview:

- Objective: Integrate basic nutrition psychoeducation into psychiatric care
- Who is this handout for? Individuals with severe mental illness or schizophrenia spectrum disorders (SSD) during periods of relative clinical stability. This tool has been developed to be delivered by a health care professional without formal nutrition training. Introduce this handout when you would normally discuss health behaviours such as sleep, substance use or physical activity.
- Reference for handout content: Aucoin M, LaChance L, Cooley K, Kidd S. Diet and psychosis: a scoping review. Neuropsychobiology. 2020;79(1-2):20-42.

Suggested Agenda:

Page 1

- Ask the patient for permission to discuss their diet and provide some basic rationale for doing so. You could read the introductory statement. If the patient declines, consider asking again at a later date.
- Ask "Why is healthy eating important to you?" to increase motivation and engagement.
- As you review the 5 recommendations, prompt the patient to circle options that appeal to them.
- Provide rationale for recommendations as per the table below if indicated.

Page 2

- Sample plate: This is a visual representation of the recommended relative proportions of different food categories in an ideal meal.
- Sample meals: Review sample meals with patient.
- Prompt patient to generate an idea of a meal that they could try based on the sample plate or sample meals. Record this on the handout.
- Healthy eating doesn't need to cost more: Discuss how spending $10 could allow them to include several items from the list. Consider pointing out the relatively increased cost of processed, convenience and restaurant foods. Direct patient to a social worker if food insecurity is a concern.
- My goals:
  - *Habit I would like to continue*: Encourage patient by acknowledging and celebrating small successes to increase motivation and self-efficacy.
  - *Changes I would like to make*: Goal setting has been shown to increase accountability and likelihood of success. Consider choosing one of the 5 recommendations from page 1 as a goal.
  - *Who can help me achieve these goals?* This prompt serves to help mobilize others who can support, facilitate and hold the patient accountable to dietary change. Consider family members, friends, case manager, family doctor, dietician, social worker, nurse, OT or others.
- Closing Statement: When possible, aim to keep messaging positive, celebrate small victories and provide encouragement.

Practical Considerations:

- Pace yourself: Implementing dietary changes is a gradual process and will not occur after one session. Consider setting one goal in the first session and revisiting the topic of nutrition at later times to follow up on the goal and make additional changes.

- Behaviour modification: Draw from your existing skills in behaviour modification. If you have knowledge of behavioural activation, motivational interviewing or another approach, remember they can be applied to dietary change as well.

- Cultural sensitivity: Consider asking patients about their culture's traditional foods. Many meals and recipes can be adapted to use grains, meat, bread-products, vegetables and spices from different cultures.

- Therapeutic diets: A small body of literature supports special diets as a therapeutic option for SSD. If patients are interested in gluten-free or ketogenic diets, please refer them to a nutrition professional.

    - Kelly DL, Demyanovich HK, Rodriguez KM, Cihakova D, Talor MV, McMahon RP, Richardson CM, Vyas G, Adams HA, August SM, Fasano A. Randomized controlled trial of a gluten-free diet in patients with schizophrenia positive for antigliadin antibodies (AGA IgG): a pilot feasibility study. Journal of psychiatry & neuroscience: JPN. 2019 Jul;44(4):269-76.
    - Sarnyai Z, Palmer CM. Ketogenic Therapy in Serious Mental Illness: Emerging Evidence. International Journal of Neuropsychopharmacology. 2020 Jul;23(7):434-9.

- Dietary assessment: If you are interested in conducting a basic dietary assessment, consider asking any of the following questions prior to reviewing the 5 recommendations on page 1:
    - What is your usual pattern of meals and snacks? Do you cook at home or buy prepared foods?
    - Take me through a typical day of eating.
    - Do you avoid any foods?
    - How many servings of vegetables do you eat per day?
    - How many times per week do you eat fish or seafood?
    - What beverages do you usually drink? (look for hidden sources of sugar)

- Portion Sizes: For guidance, search "Zimbabwe Hand Jive"

Table 1: Evidence supporting dietary recommendations and proposed mechanisms of action

Recommendation	Supporting Evidence	Mechanism of Action
Choose complex carbs	-Observational studies have reported higher intake of refined carbohydrates and lower intake of fibre in individuals with SSD	-Complex carbohydrates are higher in fibre<br>-A diet rich in fibre can improve blood glucose regulation (Level 1) and support a healthy gut microbiome composition (Level 1)<br>-A healthy gut microbiome can support mental and physical health via the gut-brain axis and by modulating systemic inflammation; probiotic supplementation in patients with SSD has shown benefit (Levels 2)
Choose healthy fats like olive oil and sources of omega-3	-Observational studies have reported low levels of essential fatty acids such as omega-3 in individuals with SSD<br>-Intervention studies of omega-3 fatty acid supplements have demonstrated efficacy in early psychosis (Level 2), clinical high risk of psychosis (Level 2), and metabolic outcomes in chronic schizophrenia (Level 1)	-Dietary fatty acids are incorporated into neuronal cell membranes and play important roles in modulating membrane fluidity and signal transduction<br>-Inflammation is relevant to the pathophysiology of SSD. Omega-3 fatty acids have anti-inflammatory properties.
Reduce highly processed foods and sugar	-Observational studies have reported higher intake of processed food and lower diet quality in individuals with SSD<br>-Intervention studies aimed at improving diet quality (as part of a multi-component interventions targeting health behaviours) have shown benefit for mental health outcomes in individuals with SSD (Level 2)	-Processed foods tend to be higher in sugar and lower in fibre and micronutrients. A high intake of processed foods can displace more nutrient-dense foods from the diet.
Add vegetables & fruits to meals and snacks	-Observational studies have reported low intake of vegetables and fruit in individuals with SSD<br>-Observational studies have reported low levels of vitamin C and folate in individuals with SSD.<br>-Intervention studies of folic acid in individuals with SSD have reported reduced negative and general symptoms of schizophrenia (Level 1)	-Vegetables and fruits provide micronutrients such as folate and vitamin C as well as fibre, antioxidants and phytonutrients<br>-Human and animal studies suggest that improvements in SSD symptoms in response to vegetable or phytonutrient supplementation are mediated by a reduction in inflammation and oxidative stress
Eat protein-rich foods throughout the day	-Intervention studies of essential amino acid supplements have reported benefit on positive, negative, general (Level 1) and cognitive (Level 2) symptoms of SSD<br>-Observational studies have reported low levels of zinc and vitamins B12 and B6 in individuals with SSD<br>-Intervention studies of zinc supplements have reported benefit on positive and negative symptoms of SSD (Level 2)<br>-Intervention studies of vitamins B6 and B12 have reported benefit on general symptoms of SSD (Level 1)	-Insufficient dietary protein consumption leads to deficiency in essential amino acids which cannot be produced by the body. Protein rich foods are also important sources of zinc, vitamin B12 and vitamin B6.<br>-Essential amino acids serve important biological roles such as neurotransmitter synthesis and NMDA receptor modulation

Legend:
Level of evidence 1 (highest) to 5 (lowest) ranked according to OCEBM Levels of Evidence Working Group*. "The Oxford Levels of Evidence 2". Oxford Centre for Evidence-Based Medicine. https://www.cebm.ox.ac.uk/resources/levels-of-evidence/ocebm-levels-of-evidence
